# Self-reported hypoglycemia and associated factors among patients living with T1D s at University of Gondar Comprehensive Specialized Hospital, Northwest, Ethiopia: a cross-sectional study

**DOI:** 10.3389/fcdhc.2025.1320610

**Published:** 2025-03-05

**Authors:** Yilkal Belete Worku, Masho Tigabe Tekle, Abaynesh Fentahun Bekalu, Mulat Belay Simegn

**Affiliations:** ^1^ Department of Internal Medicine, School of Medicine, College of Medicine and Health Sciences, University of Gondar, Gondar, Ethiopia; ^2^ Department of Clinical Pharmacy, School of Pharmacy, College of Medicine and Health Sciences, University of Gondar, Gondar, Ethiopia; ^3^ Department of Public Health, College of Medicine and Health Sciences, Debre Markos University, Debre Markos, Ethiopia

**Keywords:** self, reported, hypoglycemia, associated, factors

## Abstract

**Background:**

Hypoglycemia is a major public health problem that negatively influences blood glucose control in the treatment of type 1 diabetes. It has more severe clinical and economic effects in patients living with T1D patients. However, real-world clinical evidence of reported hypoglycemia is limited. Thus, the purpose of the study was to determine the prevalence of self-reported hypoglycemia and its associated factors among patients living with T1Dat the University of Gondar Comprehensive Specialized Hospital (UOGCSH).

**Methods:**

A prospective hospital-based cross-sectional study was conducted among patients living with T1D attending the ambulatory clinic of UOGCSH from November 1, 2021, to April 30, 2022. To select the study participants, a convenient sampling technique was used. Multivariable binary logistic regression was used to identify predictors of self-reported hypoglycemia. A P-value < 0.05 was considered statistically significant and reported as a 95% Confidence Interval (CI).

**Results:**

A total of 216 patients living with T1D (mean age: 50.91 ± 18.98 years) were included. The mean duration of DM diagnosis and insulin use were 9.41 ± 8.00 and 7.10 ± 6.00 years, respectively. Self-reported hypoglycemia was prevalent among 86.6% (95% CI: 82.1-91.0) of the study participants, with 69% experiencing non-severe and 31% experiencing severe hypoglycemia. More than half of the patients, 122 (56.5%), reported experiencing four or more (≥ 4) episodes of hypoglycemia. Knowledge of insulin self-administration, specifically a low level of knowledge (AOR=4.87; 95% CI: 1.55-15.26), was significantly associated with self-reported hypoglycemia. The majority of patients living with T1D, 155 (71.8%), had impaired awareness of hypoglycemia.

**Conclusion:**

Self-reported hypoglycemia was considerably high among Patients living with T1D. Knowledge of insulin self-administration, specifically at a low level, was associated with an increased risk of reported hypoglycemia. Thus, continued health education of Patients living with T1D regarding insulin self-administration and awareness of hypoglycemia symptoms is necessary to prevent further complications.

## Introduction

Diabetes mellitus (DM) is a group of metabolic diseases characterized by hyperglycemia resulting from defects in insulin secretion, insulin action, or both. Diabetes mellitus is a major cause of significant morbidity and mortality, affecting the entire world’s population. Further diabetic complications increase the likelihood of hospitalization, healthcare costs, lengthen hospital stays, and lower health-related quality of life (1–3).

Since hypoglycemia is one of the acute complications of diabetes that most Patients living with T1D fear, it represents a challenge to optimal diabetes treatment for both T1D and T2D (4). Due to the fear of hypoglycemic episodes, patients may avoid insulin therapy, resulting in poor glycemic control. The Canadian survey on self-reported hypoglycemia in patients living with T2D revealed that, due to the perceived risk of hypoglycemia, approximately a quarter of patients living with T2D intentionally miss, mistime, or reduce their basal insulin dose (5, 6).

Clinical practice guidelines explain that hypoglycemia is defined as a clinical syndrome caused by an abrupt reduction of blood glucose level to below 70 mg/dl and responsible for non-specific signs and symptoms, such as trembling, anxiety, diaphoresis, dizziness, hunger, nausea, confusion, tiredness, sleepiness, seizures, and loss of consciousness (7–9). In addition, hypoglycemia has been related to long-term poor clinical outcomes such as weight gain, lower quality of life, increased risk of cardiovascular disorders, anxiety, depression, poor adherence to treatment, frequent hospitalizations, extended hospital stays, and high mortality rates. Furthermore, hypoglycemia increases the need for healthcare resources, decreases productivity, reduces patients’ social interactions and self-confidence (10–13). A retrospective study at the Mayo Diabetes Clinic showed that reported severe hypoglycemia was associated with a 3.4-fold increased risk of mortality (12). In Ethiopia, studies have reported a high prevalence of hypoglycemia and have identified it as the main reason for the high morbidity and mortality rate (14, 15). To reduce the negative impact of hypoglycemia, a report from the work group of the American Diabetes Association and the Endocrine Society recommended that patients should be alert and recognize the signs and symptoms of hypoglycemia when plasma glucose is at or under 70 mg/dl (7). However, due to variability in patients’ age, duration of diabetes, and its complications, patients living with T1D patients differ in how much they experience hypoglycemic symptoms (16).

Studies conducted across the developed countries and in some part of Africa described that self-reported hypoglycemia among patients living with T1D as higher, with an estimated rate of 35.2% to 100%.

Further these studies indicated that factors such as being female, older age, higher body mass index, longer duration of diabetes, being unemployed, lack of education, impaired awareness of hypoglycemia, lack of knowledge about diabetes, longer duration of insulin use, and lower HbA1c were associated with a high prevalence of self-reported hypoglycemia (9, 14, 17–19). Few studies also reported that a lack of knowledge on insulin self-administration and taking a higher average daily dose of insulin were factors associated with hypoglycemia (18, 20). Despite hypoglycemic event in patients with diabetes is significantly associated with adverse clinical outcomes and increased medical costs in low resource setting countries including Ethiopia limited data is available on the real world prevalence rate of hypoglycemia. Up to the literature, in Ethiopia, only two (21, 22) studies regarding the extent of reported hypoglycemia and its associated factors among patients living with T1D were conducted and they explained that reported hypoglycemia was considerably high; 47.1% in southwest Ethiopia (21) and 86.7% in East Gojjam, Northwest Ethiopia (22). In an effort to minimize and reduce the negative impact of hypoglycemia among patients living with T1D, determining the magnitude of reported hypoglycemia and identifying of its risk factors as reported by patients is crucial. Further, in developing effective interventions which might improve management and prevention practice of hypoglycemic episodes at home, identification of patients living with T1D who are more likely to develop this complication is mandatory. In addition to filling the information gap regarding the magnitude of self-reported hypoglycemia and its associated factors at UOGCSH, it will serve as a baseline data for future studies that would be conducted in this areas. Thus, the purpose of this study was to determine the prevalence of self-reported hypoglycemia and its associated factors among patients with type 1 diabetes at UOGCSH, Northwest, Ethiopia.

## Materials and methods

### Study design and area

A prospective cross-sectional study was conducted at the ambulatory clinic of UOGCSH from November 1, 2021, to April 30, 2022. The clinic is located in Gondar town, which is 738 km away from the capital city, Addis Ababa. Currently, the hospital serves as a teaching and medical care center for Gondar and the neighboring residents at both the in-patient and out-patient levels. The facility has 6 outpatient department rooms and 21 beds for inpatient services.

### Population

The source population consisted of adult patients living with T1D who were under follow-up at the ambulatory clinic of UOGCSH. The study population included patients living with T1D who visited the clinic for follow-up during the study period and met the inclusion criteria.

### Inclusion and exclusion criteria

Being diagnosed with type 1 DM, age ≥ 18 years, using insulin therapy for more than 1 year, having at least 6 consecutive months of fasting blood sugar (FBS) readings, having a follow-up visit at the ambulatory clinic of UOGCSH from November 1, 2021, to April 30, 2022, and expressing willingness to participate in the study by providing informed oral consent were included. Pregnancy, serious illness, comorbidity with mental illness disorder, diabetes secondary to malnutrition and infection were excluded.

### Sample size determination and sampling procedure

The study sample comprised adult patients living with T1D who visited the ambulatory clinic of UOGCSH from November 1, 2021, to April 30, 2022, and met the inclusion criteria. The study participants were selected using a convenient sampling technique.

### Study variables

The dependent variable was the prevalence of self-reported hypoglycemia, defined as the percentage of patients who experienced at least two hypoglycemic symptoms within the previous six months. The prevalence of self-reported hypoglycemia was determined based on two criteria - symptoms experienced by a patient after injecting insulin and relief of the symptoms using sugar/candy/honey. Hypoglycemia was assessed by asking patients whether they had experienced at least two symptoms of hypoglycemia at two different times after injecting insulin in the past 6 months. Additionally, a hypoglycemic episode that required assistance from another person or medical help in a hospital for corrective measures was categorized as severe hypoglycemia (6). The socio-demographic characteristics of the patients (sex, age, educational status, residence, occupation, and marital status), body mass index (BMI), duration of DM, duration of insulin use, membership in the Ethiopian Diabetic Association (EDA), FBS of the last six follow-up visits, type of insulin used, monitoring of blood glucose at home, self-reported possible reasons for the occurrence of hypoglycemia, awareness of hypoglycemia, knowledge, and attitude regarding insulin self-administration were considered as independent variables.

### Data collection instrument and procedures

Data was collected using a pretested interviewer-administered questionnaire by four trained nurses and one supervisor. The questionnaire was prepared by reviewing similar studies conducted previously (20, 21, 23, 24). The reliability and validity of this questionnaire was confirmed by previously conducted similar study in Ethiopia which reported that the reliability coefficient of the knowledge and attitude section of this questionnaire was found to be significant (Cronbach’s alpha: 0.77) (Hypoglycemia Among Type 1 Diabetes Patients After Insulin Use in Southwest Ethiopia). It included statements about the clinical manifestations of hypoglycemia and the immediate measures that should be taken to resolve hypoglycemia at home. Socio-demographic variables and medical history were collected through medical chart review and patient interviews. Information regarding hypoglycemia symptoms, awareness, management at home, possible reasons for occurrence, and knowledge and attitude regarding insulin self-administration were obtained through patient interviews.

### Data quality control technique

To ensure questionnaire consistency, a pre-test was conducted at the ambulatory clinic of UOGCSH two weeks before data collection, and modifications were made accordingly. Before actual data collection, training was provided to four nurses and one supervisor on the study objectives and data collection process. During the data collection period, regular supervision was conducted, and collected data were checked daily for completeness and consistency.

### Data entry and statistical analysis

Data was edited, cleaned, coded, entered into EPI DATA version 4.6.0.2, and exported to Statistical Package for Social Sciences version 25 for analysis. Descriptive analysis was used to summarize socio-demographic variables, membership in the diabetic association, monitoring of blood glucose, and type of insulin used. Variables related to hypoglycemia, such as symptoms, possible reasons, awareness, and home management, were described using descriptive statistics. Continuous variables were expressed as mean (± SD), while categorical variables were summarized as frequency (percentage). Binary logistic regression was used to determine factors associated with self-reported hypoglycemia. The Hosmer-Lemeshow goodness-of-fit test was used to assess the model’s fitness. Both crude odds ratio (COR) and adjusted odds ratio (AOR) with the corresponding 95% confidence interval (CI) were used to measure the strength of association. A p-value < 0.05 in the multi-variable regression model was considered statistically significant.

### Operational definitions

Self-reported hypoglycemia: The prevalence of hypoglycemia was determined based on two criteria - symptoms experienced by a patient after injecting insulin and relief of the symptoms using sugar/candy/honey. Hypoglycemia was assessed by asking patients whether they had experienced at least two symptoms of hypoglycemia at two different times after injecting insulin in the past 6 months. Additionally, a hypoglycemic episode that required assistance from another person or medical help in a hospital for corrective measures was categorized as severe hypoglycemia (6).

#### Good, average, and low knowledge about insulin self-administration

Patients who scored > 9-13 (> 66%), 5-8 (33.3-66.6%), and 0-4 (< 33.3%) of correct responses from knowledge questions, respectively (20).

#### Favorable attitude about insulin self-administration

Patients who scored > 6 (> 49%) on attitude questions. Unfavorable attitude refers to a person who scored less than 6 (< 50%) on attitude questions (20).

#### Insulin self-administration

The injection of insulin expected to be administered at home without assistance.

#### Awareness of hypoglycemia symptoms

Patients who answered “always” to the question “Can you feel when your blood sugar is low?” (19).

#### Unaware of hypoglycemia symptoms

Patients who answered “usually, occasionally, and never” to the question “Can you feel when your blood sugar is low?” (19).

## Results

### Socio-demographic and clinical characteristics of patients living with T1D

Between November 1, 2021, and April 30, 2022, a total of 240 Patients living with T1D visited the ambulatory clinic of UOGCSH. Of these, 216 Patients living with T1D who met the inclusion criteria were included in this study. The mean (± SD) age was 50.91 (± 18.98) years, and 111 (51.4%) were females. More than half, 131 (60.6%) of the patients had no formal education and nearly one third of them were farmers, 68 (31.5%). The median (IQR) duration of DM diagnosis was 8 (IQR: 13-6) years, while the median (IQR) duration of insulin use was 6 (IQR:10-6) years. The mean (± SD) BMI of the patients was 22.01 (± 2.47) kg/m2. The median (IQR) FBS level of the patients living with T1D was 154.5 (IQR: 210.8-102.0) mg/dl. The mean (± SD) knowledge and attitude scores of the patients living with T1D regarding insulin self-administration were 3.84 (± 1.92) and 10.49 (± 2.98), respectively. Although the majority, 162 (75.0%), of the patients had a low level of knowledge, the majority, 172 (79.6%), had a favorable attitude about insulin self-administration. In the study majority of patients, 201 (93.1%) were using NPH insulin and only 13 (6%) of patients monitor their blood glucose level at home (**Table 1A**). There was a statistically significant difference in the prevalence of self-reported hypoglycemia with regard to gender (χ2 (1, N = 216) = 4.143, p = 0.042, female = 51.34% Vs male = 48.66%) and knowledge on insulin self-administration ((χ2 (1, N = 40) = 11.552, p = 0.003, low = 78.6%, average = 15.5%, and good = 5.88%) (**Table 1B**).

**Table 1A:** Socio-demographic and clinical characteristics of patients living with T1D at UOGCSH, Northwest, Ethiopia, 2022.

Variable	Category	Frequency (%)
Gender	Male	111 (51.4)
	Female	105 (48.6)
Age (years)		
	18-29	38 (17.6)
	30-44	50 (23.1)
	45-59	53 (24.5)
	≥ 60	75 (34.7)
Residence		
	Urban	130 (60.2)
	Rural	86 (39.8)
Educational status		
	Unable to read and write	104 (48.1)
	Able to read and writ	16 (7.4)
	Primary	19 (8.8)
	Secondary	35 (16.2)
	College and above	42 (19.4)
Occupation		
	Housewife	58 (26.9)
	Farmer	61 (28.2)
	Government employee	32 (14.8)
	Non-governmental organization employee	16 (7.4)
	Private business	49 (22.7)
Body max index (kg/m^2^)		
	< 18.5	18 (8.3)
	18.5-24.9	162 (75.0)
	25-29.9	21 (9.7)
	30-34.9	15 (6.9)
Membership to the EDA	Yes	140 (64.8)
	No	76 (35.2)
Duration of DM (years)		
	≤ 8	136 (63.0)
	> 8	80 (37.0)
Duration of insulin use (years)		
	≤ 6	138 (63.9)
	>6	78 (36.1)
Type of insulin taken		
	NPH	193 (89.4)
	Mixed (NPH insulin + Regular insulin)	23 (10.6)
FBS (mg/dl)	< 70	87 (40.3)
	70-130	72 (33.3)
	≥ 131	57 (26.4)
Monitoring of blood glucose		
	Yes	18 (8.3)
	No	198 (91.7)
Knowledge about insulin self-administration		
	Low	162 (75.0)
	Average	37 (17.1)
	Good	17 (7.9)
Attitude about insulin self-administration		
	Unfavorable	44 (20.4)
	Favorable	172 (79.6)

T1D, Type 1 diabetes; UOGCSH, University of Gondar Compressive Specialized Hospital.

% = percent, EDA, Ethiopian Diabetic Association.

**Table 1B:** Comparison of prevalence of self-reported hypoglycemia among patients living with T1D based on their socio-demographic and clinical characteristics at UOGCSH, Northwest, Ethiopia, 2022.

Variable	Category	Hypoglycemia		Chi-Square (χ2)	P-value
		Yes	No		
Gender				4.143	0.042*
	Male	91	20		
	Female	96	9		
Age (years)				1.395	0.707
	18-29	32	6		
	30-44	45	5		
	45-59	44	9		
	≥ 60	66	9		
Residence				1.001	0.317
	Urban	115	15		
	Rural	72	14		
Occupation				5.239	0.264
	House wife	52	6		
	Farmer	54	7		
	Government employee	27	5		
	Non-governmental organization employee	11	5		
	Private business	43	6		
Educational status				14.916	0.005*
	Unable to read and write	98	7		
	Able to read and write	11	6		
	Primary education	13	5		
	Secondary education	28	6		
	College and above	37	5		
Membership to the EDA				0.564	0.453
	Yes	123	17		
	No	64	12		
Duration of DM (years)				3.099	0.078
	≤ 8	122	14		
	>8	65	15		
Duration of insulin use (years)				3.539	0.060
	≤ 6	124	14		
	>6	63	15		
Types of insulin taken				6.407	0.011*
	NPH insulin	171	22		
	Mixed (NPH insulin + Regular insulin)	16	7		
Monitoring of blood glucose at home using glucometer				16.254	0.000**
	Yes	177	21		
	No	10	8		
Body max index (kg/m^2^)				27.731	0.000**
	< 18.5	13	5		
	18.5-24.9	151	11		
	25-29.9	15	6		
	30-34.9	8	7		
FBS (mg/dl)				0.323	0.851
	< 70	76	11		
	70-130	61	11		
	≥ 131	50	7		
Knowledge about insulin self-administration				11.552	0.003*
	Low	147	15		
	Average	29	8		
	Good	11	6		
Attitude about insulin self-administration				0.293	0.588
	Unfavorable	37	7		
	Favorable	150	22		

^*^Statistically significant at p < 0.05. ^*^Statistically significant at p < 0.01. T1D, Type 1 diabetes; UOGCSH, University of Gondar Compressive Specialized Hospital.

### Prevalence of self-reported hypoglycemia and hypoglycemia awareness

**Figure 1:**
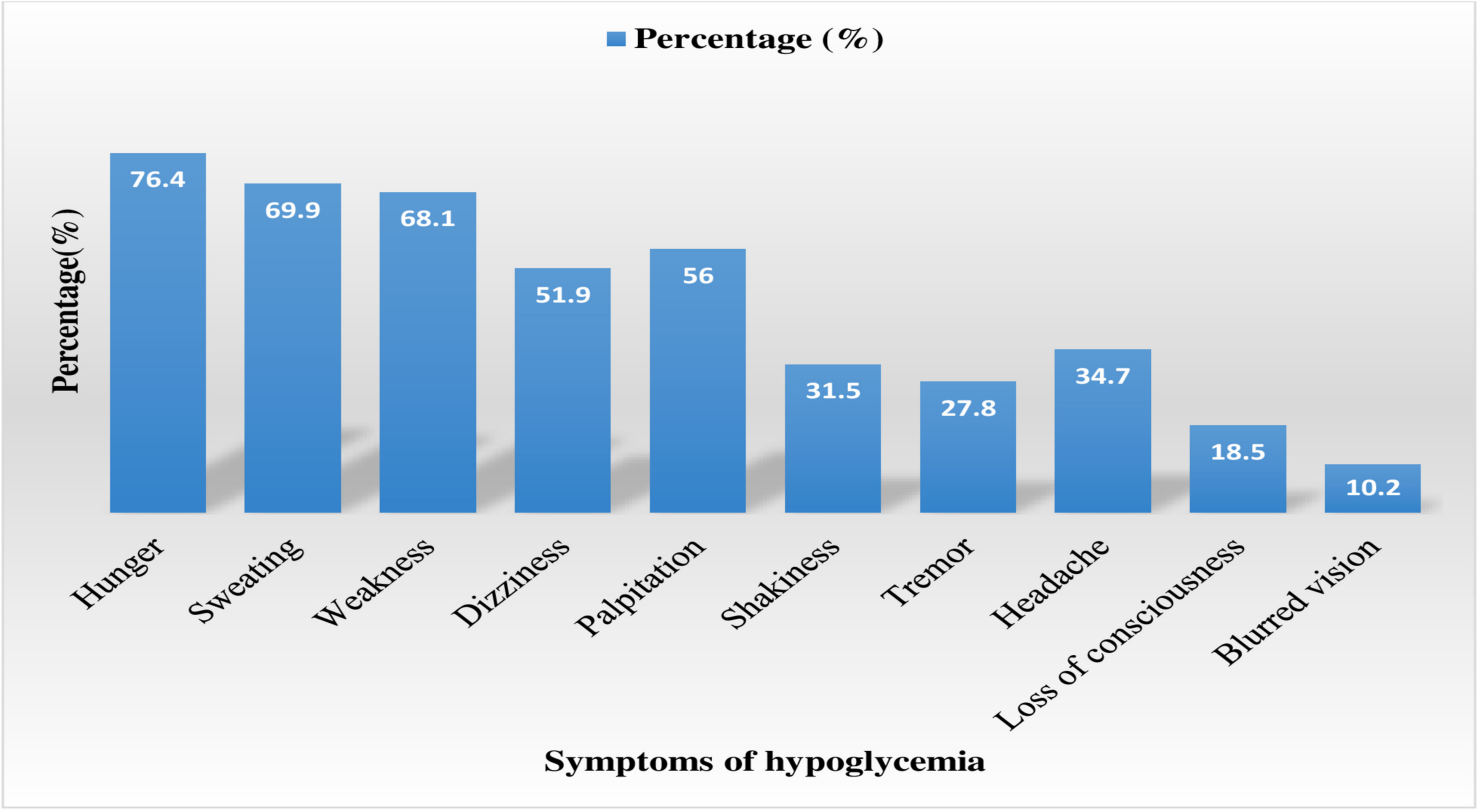
Self-reported symptoms of hypoglycemia among patients with T1D at UOGCSH, Northwest, Ethiopia, 2022. T1D, Type 1 diabetes; UOGCSH, University of Gondar Compressive Specialized Hospital.

The reported prevalence of hypoglycemic events within the last 6 months was 86.6% (95% CI: 82.1-91.0), with 69% experiencing non-severe and 31% experiencing severe hypoglycemia. More than half, 122 (56.5%), of the patients reported that they had experienced four or more (≥ 4) episodes of hypoglycemia. Additionally, only 61 (28.2%) of the patients living with T1D were aware of hypoglycemia, while the majority, 155 (71.8%), had impaired awareness of hypoglycemia. The most frequently reported symptoms of hypoglycemia were hunger 165 (76.4%), sweating 151 (69.9%), weakness 147 (68.1%), and palpitation 121 (56.0%) (**Figure 1**). Of those who developed hypoglycemia, 58 (26.9%), 35 (16.2%), and 29 (13.4%) managed it using sugar, sweet candies, and soft drinks, respectively. Delayed meal 88 (19.9%), followed by exercise 55 (25%) and taking a higher dose of insulin 49 (22.7%), were the most common precipitating factors responsible for hypoglycemic episodes (**Table 2**).

**Table 2:** Reasons contributing to hypoglycemia among patients living with T1D at UOGCSH, Northwest, Ethiopia, 2022.

Reasons contributing to hypoglycemia	Category	Frequency (%)
Delayed meal	Yes	88 (40.7)
	No	128 (59.3)
Exercise	Yes	54 (25.0)
	No	162 (75.0)
Taking higher dose of insulin	Yes	49 (22.7)
	No	167 (77.3)
Taking alcohol with food	Yes	10 (4.6)
	No	206 (95.4)
Taking alcohol without food	Yes	13 (6.0)
	No	203 (94.0)

T1D, Type 1 diabetes; UOGCSH, University of Gondar Compressive Specialized Hospital.

### Factors associated with self-reported hypoglycemia

Multivariable logistic regression analysis indicated that knowledge of insulin self-administration, specifically a low level of knowledge (AOR=4.87; 95% CI: 1.55-15.26), was a predictor variable for self-reported hypoglycemia. Therefore, the study’s findings suggested that patients with a low level of knowledge regarding insulin self-administration were 4.87 times more likely to develop hypoglycemia compared to patients with good knowledge (AOR=4.87; 95% CI: 1.55-15.26) (**Table 3**).

**Table 3:** Bi-variable and multi-variable logistic regression analysis of self-reported hypoglycemia among patients living with T1D at UOGCSH, Northwest, Ethiopia, 2022.

Variables	Category	Self-reported hypoglycemia		COR (95%CI)	AOR (95%CI)	P-value
		Yes	No			
Sex	Female	96	9	2.34 (1.02,5.42)	2.02 (0.86,4.79)	0.108
	Male	91	20	Ref	Ref	
Knowledge of insulin self-administration	Low	147	15	5.35 (1.73,16.51)	4.87 (1.54,15.26)	0.007*
	Average	29	8	1.977 (0.558,7.011)	2.01 (0.56,7.22)	0.286
	Good	11	6	Ref	Ref	

T1D, Type 1 diabetes; UOGCSH, University of Gondar Compressive Specialized Hospital. ^*^Statistically significant at p < 0.05, Ref, Reference category; COR, Crude Odd Ratio; AOR, Adjusted Odd Ratio; CI, Confidence Interval.

## Discussion

Hypoglycemia is a common complication and often impossible to eliminate from the lives of patients with T1D (25). However, intensive glycemic control can prevent and slow the progression of microvascular and macrovascular complications in these patients (26). This study revealed that the prevalence of self-reported hypoglycemia was 86.6% (95% CI: 82.1-91.0). The multivariable logistic regression analysis showed a significant association between a low level of knowledge regarding insulin self-administration and the high prevalence of self-reported hypoglycemia.

The mean FBS level of the patients living with T1D was 168.83 (± 130.45) mg/dl. This finding is comparable with a study in Jimma and Addis Ababa, Ethiopia, where the mean FBS was 171 (± 63) mg/dl (27). However, this finding was higher than the patients living with T1D goals recommended by several guidelines (6, 28). More than two-thirds, 145 (67.1%), of the patients had poor glycemic control which was consistent result reported by a study in Addis Ababa, Ethiopia, where 65% of patients had poor glycemic control (29). The practice of self-monitoring blood glucose levels at home was low, with only 13 (6%) of the participants engaging in this practice. This finding was also similar to previous study in Addis Ababa (30). This low rate could be related to the financial capacity of the patients to afford glucometers and test strips in the study areas.

In the present study prevalence of self-reported hypoglycemia among patients with type 1 diabetes was 86.6% (95% CI: 82.1-91.0). This finding was consistent with rates reported in Tikur Anbessa Specialized Hospital, Ethiopia (88%) [27], Debre Markos Referral Hospital, Ethiopia (86.7%) [24], and Turkey (84.1%) [19]. However, it was higher than rate reported in Nigeria (35.2%) [15] On the other hand, the prevalence of self-reported hypoglycemia in this study was lower than the findings from an international survey in nine countries (97.4%) (23), Philippine (100%) (16), and Singapore (100%) (24).

The variable prevalence of hypoglycemic episodes in this study and other studies can be attributed to differences in the epidemiological characteristics of the study population, such as demographics, ethnicity, religious variations, and the operational definitions and methods of reporting. Other contributing factors could include differences in awareness about hypoglycemia among participants, study settings, duration of DM in the study population, and a wide spectrum of therapeutic regimens used in the management of DM. Additionally, the variability in the inclusion and exclusion criteria of participants in different studies and differences in self-monitoring practices of study participants could also play a role. The non-specific nature of hypoglycemia symptoms might also contribute to differences in reported prevalence rates.

In this study, 31% of the patients experienced severe hypoglycemia. This rate is higher than what has been reported Turkey (15.5%) (24). This difference may be explained by the fact that only a small proportion of patients living with T1D (6%) practiced self-monitoring of blood glucose in our study.

The study also revealed that only a quarter (28.2%) of patients with type 1 diabetes were aware of the symptoms of hypoglycemia. This is in line with rates of awareness reported in Tikur Anbessa Hospital, Ethiopia (28.4%) (29). However, it is lower than rates reported in Turkey (83.4%) (24), Germany (65%) (25), and Spain (55%) (26). The lower prevalence of awareness of hypoglycemia in this study might be explained by the high prevalence of hypoglycemic episodes in the study population, leading to hypoglycemia unawareness through hypoglycemia-associated autonomic failure (31). Additionally, the longer duration of DM could be another contributing factor.

The study further revealed that hunger (76.4%) was the most reported symptom of hypoglycemia, followed by sweating (69.9%), weakness (68.1%), dizziness (51.9%), and palpitations (44%). This is consistent with the findings of similar studies in which hunger, sweating, weakness, and dizziness were the most frequently reported hypoglycemia symptoms (15–17, 23). Delayed meal (40.7%) and exercise (25%) were the most common precipitating factors responsible for hypoglycemic episodes in this study. This finding is supported by several studies that have explained that hypoglycemia is often precipitated by delayed meals and heavy physical activity (29).

The findings of this study revealed that patients who had low level of knowledge regarding insulin self-administration were 4.87 times more likely to develop hypoglycemia compared to patients who had good knowledge. The findings of the present study showed that low level of knowledge regarding insulin self-administration was a predictor variable for self-reported hypoglycemia. A study in Southwest, Ethiopia and Turkey also explained that lack of knowledge on insulin self-administration was factor associated with high rate of reported hypoglycemia (21, 24). In spite of its prospective nature this study has limitations, including its single-population focus and the fact that it was conducted in a single health facility, which may limit the generalizability of the results.

## Conclusion

The present study demonstrated a high self-reported prevalence of hypoglycemia among patients living with T1D. Additionally, having a low level of knowledge regarding insulin self-administration was significantly associated with a high prevalence of self-reported hypoglycemia. Therefore, health education for patients living with T1D on insulin administration and the detection and management of hypoglycemia symptoms at home is necessary.

## Data Availability

The raw data supporting the conclusions of this article will be made available by the authors, without undue reservation.
